# MCQs in-training examination scores of the surgical residency program in Thailand: the relationship between medical school vs public health-based training institutions

**DOI:** 10.1186/s12909-024-06063-0

**Published:** 2024-09-27

**Authors:** Saritphat Orrapin, Ob-Uea Homchan, Narain Chotirosniramit, Tidarat Jirapongcharoenlap, Chagkrit Ditsatham

**Affiliations:** 1 Department of Surgery, Faculty of Medicine, Thammasat University, Thammasat University Hospital, Pathum Thani, Thailand; 2 Training and Examination Board of Certification in Fellowship of Surgery of the Royal College of Surgeons of Thailand, Bangkok, Thailand; 3 Department of Surgery, Faculty of Medicine, Chiang Mai University, 110 Intavaroros Road, Amphoe Muang, Chiang Mai, 50200 Thailand; 4 Clinical Surgical Research Center, Faculty of Medicine, Chiang Mai University, Chiang Mai, Thailand

**Keywords:** MCQs, Assessment, In-training, Examination, Surgery, Residency

## Abstract

**Background:**

This study aims at investigating and evaluating the categorical knowledge of residency using an internet-based examination.

**Methods:**

All in-training examinees from 32 Thailand’s general surgery residency training institutions participating in the online examination. One hundred fifty Multiple Choice questions (MCQs) were selected for this examination from a pool of previous MCQs used for board certification examinations. Baseline chracteristic of the examinee including residency year, training institution (medical schools and public health-based training institutions), regional-based area of the institution, overall test score, scoring by subcategory, total time to complete the examination, and the length of accredited as a training centre time were collected and analysed.

**Results:**

Total 613 examinees. The mean total score of 1st and 3rd year residency of a public health hospital institution differed from those with medical school-based training. On average, the scores in 4 out of 10 categories of residency from medical school training institutions were higher. However, residency from institutions with over 7 years of experience tended to score higher in the Liver, Biliary and Pancreas category.

**Conclusions:**

The average scores of MCQs exams which reflect the medical knowledge for general surgical residency training at medical schools do not significantly differ from those of individuals undergoing public health training. Additionally, the mean scores of MCQs exams did not differ between high-experience training institutions and recent accredited training centers.

## Background

Medical students in both the UK and the USA have been assessed using MCQs in examinations for over 20 years and they are also commonly used in postgraduate exams [1, 2]. The current debate surrounding MCQs focuses primarily on the pros and cons of different types of questions used in these tests [3]. Assessment methods, including method in question, have their own unique strengths and limitations [4]. In the past, studies have found that the results of evaluating knowledge through MCQs are good when compared to other methods of assessment [4–6]. This online, nationwide in-training MCQ surgical examination in Thailand was created by the RCST. The purpose of this examination is to enable surgical residents to self-evaluate and improve their knowledge in each category, as well as to prepare for the Thai Board of Surgery Qualifying Exam by the RCST in the final year of their training program. With the COVID-19 pandemic, the individual computer-based examination platform became especially convenient due to travel restrictions and social distancing policies.

The General Surgery Residency Training Program of Thailand was established in 1980 [7]. The duration of the surgical training was 4 years. To achieve certification in the board examination, candidates need to pass all three parts of the exam: (1) Multiple choice questions (MCQs), (2) Modified Essay Questions (MEQs), and (3) an oral examination. The multiple-choice questions (MCQs) for this examination were carefully chosen from a well-established pool of subjects previously used in board certification exams. These questions were selected by the surgical faculty from 32 general surgery residency training institutions across Thailand. The surgical faculty included both medical-school and public health hospital staff. The faculty members involved are recognized experts in their respective fields, with specialized qualifications in 10 general surgery subspecialties. These subspecialties include esophageal, gastric, and small bowel surgery; head and neck surgery, endocrineprocedures; hepatobiliary and pancreatic (HPB) surgery; vascular surgery; trauma surgery; plastic surgery, burns management; breast surgery; skin and soft tissue surgery; and colorectal and appendiceal surgery. Additionally, the subspecialties encompass areas outside general surgery, such as neurosurgery, cardiovascular thoracic surgery, urologic surgery, pediatric surgery, and bariatric surgery. The selected MCQs were rigorously validated and accredited by the committees of the Training and Examination Board of Certification in Fellowship of Surgery at the Royal College of Surgeons of Thailand (RCST) in Bangkok, Thailand [8, 9].

The main surgical training institutions approved by the RCST can be divided into two categories: medical school-based training institutions and public health hospital-based (regional or provincial hospital) training institutions. Medical school-based training institutions tend to have higher educational resources, such as surgical simulations, library access, and more up-to-date research literature, due to the support system from the university or college. The surgical staff who traingeneral surgical residents typically have obtained advanced training or subspecialties beyond general surgery. For example, the surgical staff of medical school-based training institutions acquired specialist training abroad inhepatopancreatic and biliary surgery, colorectal, vascular surgery, endocrinologic, breast, robotic surgery, oncologic surgery, etc. Additionally, medical school-based training institutions frequently have more academic conferences than public health hospitals, allowing surgical residents to easily participate in these activities. In contrast, public health hospitals typically handle a higher number of cases and more common surgical diseases, especially in tertiary care centers under the public health system, compared to medical school-based training institutions. The surgical staff who train general surgical residents usually have extensive experience in caring for general surgical disease patients. This difference may lead to variations in experience and medical knowledge between residents trained in medical school-based and public health hospital-based institutions. These training institutions are located throughout Thailand, across the Central, Northern, Eastern, Northeastern, and Southern regions. Some institutions have been accredited to deliver the training program for a long time, while others have only recently been approved. The training institutions with more than seven years of experience in surgical residency training were re-accredited under the World Federation of Medical Education (WFME) for general surgery in 2022 by the Training and Examination Board of Certification in Fellowship of Surgery of the RCST. Therefore, for this study, the training institutions were categorized as high-experience training centers and recently accredited training centers based on a seven-year cut-off, which may affect the trainees’ knowledge and experience [9].

There is significant variation in patient characteristics, resources, and trainers’ experience among the 32 institutions that provide general surgery residency training in Thailand. The types of surgical procedures and the level of experience at these institutions are influenced by factors such as common surgical diseases local occurence, endemic diseases, culture, occupation, and the social and economic status of the regions they serve. Trainers’ experience and resources tend to be more substantial at medical school-based training institutions, particularly those with more than seven years of experience. As previously mentioned, these differences impact both the knowledge and surgical skills acquired by residents during their training at each institution [8].

The information collected in the database included the examinee’s sex, residency year, training institution, overall test score, subcategory scores, total time to complete the examination, the location of the institution, and the length of time the institution had been accredited as a training center. The data were then divided into two groups to facilitate comparison: examinees from medical schools and those from public health-based training institutions. MCQs are commonly used in postgraduate medical examinations because they are time-efficient, highly reliable, and easily standardized [10]. The main competencies under the outcome-based training system of WFME in the general surgery curriculum of RCST include patient care, medical knowledge, practice-based learning and improvement, interpersonal and communication skills, professionalism, and system-based practice [9]. The medical knowledge is the important part of main competency which involving to basic science, epidemiological and clinical, as well as the application of this knowledge to surgical patients [11]. The University of Maryland, Medical Center (UMMC) established the Accreditation Council for Graduate Medical Education (ACGME) Common Program Requirements, which mandate that every residency/fellowship program insists on its residents acquiring medical knowledge. It is mandatory programs to define the specific knowledge, skills and attitudes of residents to demonstrate competency in Medical Knowledge for surgical residents [11]. Well-constructed MCQs enable the evaluation of this competency. However, it can be challenging to design MCQs that effectively assess the application of knowledge and the competence of surgical residents. To evaluate medical knowledge in surgical residency, application-based MCQs are more appropriate for assessing understanding of surgical conditions, decision-making, and critical thinking in surgical patients. Most medical schools and residency training programs use case-based and application-based MCQs as vital components in the evaluation of surgical trainees and practicing surgeons [12]. They are less likely to developbasic recall MCQs, which are a poorevaluation of medical knowledge and other competencies. The American College of Surgeons created the Surgical Education and Self-Assessment Program (SESAP^®^) which is a case-based question and premier educational resourcepromoting excellence and expertise for practicing surgeons in the United States of America (USA) [1]. The RCST has developed an internet-based online examination using case-based and application MCQs for residency self-preparation, designed to evaluate individual knowledge. The examination consists of 150 items across different categories based on body systems. This study aims to determine the relationship between system-based MCQ scores in surgical diseases, which reflects the medical knowledge of trainees, and the type of training institution, either medical school-basedor public health hospital-based(regional or provincial). Additionally, the study compares medical knowledge between high-experience training institutions and recently accredited institutions by assessing the impact of a 7-year duration of surgical residency training.

## Gap of knowledge

Assessing knowledge through MCQ exams is a reliable method of evaluating performance. During the COVID-19 pandemic, online exams provide assessors with the convenience of conducting assessments without the need for travel or gatherings. The exam results can also be categorized, enabling examinees to review areas where they scored lower and work on improving themselves.

## Methods

### Data collection

This retrospective cohort study collected data from a database, including the sex of the examinee, residency year, training institution, overall test score, subcategory scores, total time to complete the examination, location of the institution, and the length of time the institution had been accredited as a training center. The independent variables were then categorized to enable data comparisons as follows: [1] medical school-based training versus public health hospital-based training institutions; [2] the region of the training center, including Central, Northern, Eastern, Northeastern, and Southern regions of Thailand; and [3] high-experience training centers versus recently accredited training centers, based on a 7-year cut-off for this study. The primary outcome, or dependent variable, was the mean score of the MCQ examination. Online examination scores from 613 examinees participating in the examination from 32 training institutions were collected.

### Study participants

General surgical residency students from the 1st to 4th year who were in training under the RCST curriculum as of May 7th, 2022, were included in the study to participate in the MCQ examination. Non-RCST general surgery residents, such as those from other countries who choose to study at a training institution in Thailand, were not permitted to participate in the MCQ examination.

### Selection of multiple-choice questions (MCQs)

Case-based and application MCQs were randomly selected for this examination from a pool of previous questions used in board certification exams. A total of 150 MCQs, each with five answer options, were included. The questions were equally divided into 10 categories: (1) Esophageal, gastric, and small bowel surgery; (2) Head and neck (thyroid and parathyroid) surgery; (3) Hepatobiliary and pancreatic (HPB) surgery; (4) Vascular surgery (peripheral arteries and aorta, venous system); (5) Trauma (abdominal, chest, neuro, and vascular trauma); (6) Plastic surgery and burns; (7) Breast surgery; (8) Skin and soft tissue surgery; (9) Colorectal and appendix surgery; and (10) Non-general surgery subspecialties (neurosurgery, cardiovascular thoracic surgery, urologic surgery, pediatric surgery, and bariatric surgery).

These 10 categories were determined by the committees of the Training and Examination Board of Certification in Fellowship of Surgery of the RCST and are documented in Thailand’s Surgical Residency curriculum under the World Federation of Medical Education (WFME) standards for general surgery in 2022. The MCQs underwent a pilot test, and their internal consistency was verified by the committee. The MCQ examination is an internet-based online assessment designed to evaluate individual knowledge, summarizing the examinee’s characteristics, institution, overall scores, and categorized scores using a highly reliable computer-based analysis. The total time allocated for the test is approximately 180 min, after which the system automatically logs out. Consequently, all surgical residents mean scores were measured under the same conditions and at the same time, ensuring no bias.

### Statistical analysis

Descriptive statistics were used to summarize categorical variables as frequencies and percentages. For continuous variables, if the data were normally distributed, the mean and standard deviation (SD) were reported; if the data were non-normally distributed, the median and interquartile range (IQR) were used. Regarding inferential statistics, Fisher’s exact test was applied to compare proportions between two groups, and the student’s t-test was used to compare means between two institutions. If the data were normally distributed, Analysis of Variance (ANOVA) was employed to assess differences across institutional years and residency years. However, if the data were not normally distributed, the Kruskal-Wallis test was used instead. To analyze the correlation between two groups, subgroup analysis was performed to investigate factors that may be associated with the score, such as the duration of training, subject-based category, and residency year. Statistical analyses were conducted using Stata/SE version 16 for Mac (StataCorp, College Station, TX, USA).

## Results

A total of 613 surgical residency examinees completed the examination, with 65% being male. The average overall time to finish the examination did not significantly differ between medical school-based surgical training institutions and public health hospital institutions (169.37 min vs. 170.55 min, *p* = 0.325). However, when subdivided by residency year, first-year residents from public health hospital training institutions took significantly longer to complete the examination compared to those from medical school training institutions (172.78 min vs. 167.25 min, *p* = 0.011) (Table 1).

**Table 1:** Characteristic of online surgical in-training examinators

Parameters	Medical school surgical-board training institution (*N* = 340)	Public health hospital surgical-board training institution (*N* = 273)	*p*-value
**Gender**,** n (%)**			0.607
Male	227 (66.76)	176 (64.47)	
Female	113 (33.24)	97 (35.53)	
**Residency year**,** n (%)**			0.503
1^st^ year Residency 2^nd^ year Residency 3^rd^ year Residency 4^th^ year Residency	93 (27.35) 85 (25.00) 93 (27.35) 69 (20.29)	77 (28.21) 80 (29.30) 71 (26.01) 45 (16.48)	
Average time to finished (mins), mean (SD)	169.37 (14.73)	170.55 (14.74)	0.325
**Examination (mins)**,** mean (SD)**			
1^st^ year Residency 2^nd^ year Residency 3^rd^ year Residency 4^th^ year Residency	167.25 (16.91) 171.86 (10.67) 173.28 (9.25) 163.90 (19.33)	172.78 (9.35) 172.01 (12.83) 170.48 (16.17) 164.24 (20.67)	0.011 0.933 0.164 0.928
**Total score**,** mean (SD)**			
1^st^ year Residency 2^nd^ year Residency 3^rd^ year Residency 4^th^ year Residency	45.89(7.24) 50.26(8.52) 55.58(6.96) 59.50(6.93)	43.73(6.31) 48.05(9.10) 52.88(8.59) 58.60(6.73)	0.042 0.109 0.028 0.495
**Scored by categories**,** mean (SD)**			
Esophagus, small bowel, stomach	47.43 (12.79)	46.83 (11.76)	0.550
Head and neck thyroid parathyroid surgery	45.68 (26.87)	43.86 (27.69)	0.410
Hepatobiliary and Pancreatic surgery	59.09 (17.95)	54.75 (19.02)	0.004
Peripheral arteries and aorta, venous system	56.24 (14.58)	49.95 (15.66)	< 0.001
Trauma of abdomen, chest, neuro, vascular	48.45 (13.85)	48.51 (13.41)	0.954
Plastic surgery and burn	56.30 (18.74)	53.34 (21.56)	0.071
Breast surgery	53.16 (16.25)	48.91 (17.27)	0.002
Subspecialty	50.85 (18.45)	49.72 (19.08)	0.456
Skin soft tissue	57.35 (21.92)	59.05 (23.21)	0.354
Colorectal and appendix	46.10 (14.91)	43.53 (15.44)	0.037

The mean total scores for first- and third-year residents from public health hospital institutions were significantly lower than those from medical school-based training programs. Specifically, first-year residents had a mean total score of 43.73 compared to 45.89 for medical school residents (*p* = 0.042), and third-year residents had a mean score of 52.88 compared to 55.58 (*p* = 0.028) (Table 1).

In the categories of Hepatobiliary and Pancreatic surgery, Peripheral arteries and aorta, Venous system, Breast surgery, and Colorectal and appendix surgery, the overall mean scores were significantly higher for residents from medical school institutions.

Table 2 compares each year of residency based on specific categories. It shows that in the first year, residents from medical schools scored higher than those from public health institutions in the vascular artery, aorta, and vascular veins categories (mean scores 51.4 vs. 44.75, *p* = 0.003), as well as in the colorectal and appendix categories (mean scores 39.53 vs. 35.34, *p* = 0.035). In the head and neck, breast, skin and soft tissue, and subspecialty categories, the scores of residents from public health institutions were not significantly different from those of medical school residents. In the second year of residency, statistically significant differences were observed in the vascular and breast sections (*p* = 0.01 and *p* = 0.001, respectively). While other groups did not show statistically significant differences, third-year residents from medical schools scored significantly higher than those from public health institutions in the Hepatobiliary and Pancreatic (HPB) and vascular sections (*p* = 0.009 and *p* < 0.001, respectively). Conversely, the public health group scored higher in the esophagus, small bowel, stomach, trauma, and subspecialty sections. Among fourth-year residents preparing for the oral examination, the only significant difference was in the skin and soft tissue category, where public health residents scored higher (67.56 points) compared to those from medical schools (59.13 points, *p* = 0.03).

**Table 2:** Comparison scoring by institutions year-by-year residents

Parameters	Medical school surgical-board training institution (*N* = 340)	Public health hospital surgical-board training institution (*N* = 273)	*p*-value
	Mean (SD)	Mean (SD)	
**1** ^**st**^ **year residency**			
Esophageal, gastric and small bowel surgery	43.75 (11.93)	41.58 (10.73)	0.220
Head and neck thyroid parathyroid surgery	36.90 (26.57)	38.35 (25.80)	0.721
Hepatobiliary and Pancreatic surgery	49.09 (15.83)	44.25 (17.09)	0.058
Vascular artery and aorta, Vascular veins	51.40 (15.17)	44.75 (13.58)	0.003
Trauma of abdomen, chest, neuro, vascular	44.50 (13.70)	43.87 (12.89)	0.762
Plastic surgery and burn	49.53 (17.15)	44.75 (21.30)	0.106
Breast surgery	42.61 (14.92)	44.66 (17.36)	0.408
Subspecialty	48.48 (17.13)	50.17 (19.26)	0.546
Skin soft tissue surgery	51.40 (23.11)	51.95 (23.90)	0.879
Colorectal and appendix surgery	39.53 (13.07)	35.34 (12.40)	0.035
**2** ^**nd**^ **year residency**			
Esophageal, gastric and small bowel surgery	45.53 (14.06)	46.61 (11.45)	0.590
Head and neck thyroid parathyroid surgery	44.57 (25.53)	42.45 (26.39)	0.608
Hepatobiliary and Pancreatic surgery	53.53 (17.75)	3.68 (17.81)	0.958
Vascular artery and aorta, Vascular veins	54.33 (13.46)	48.56 (15.05)	0.010
Trauma of abdomen, chest, neuro, vascular	48.85 (13.46)	48.42 (13.30)	0.838
Plastic surgery and burn	51.84 (19.15)	51.11 (20.67)	0.814
Breast surgery	51.67 (15.57)	43.37 (17.30)	0.001
Subspecialty Skin soft tissue surgery Colorectal and appendix surgery	49.46 (20.21) 58.35 (21.65) 41.64 (13.62)	46.57 (17.20) 60.50 (23.81) 41.30 (14.85)	0.324 0.545 0.879
**3** ^**rd**^ **year residency**			
Esophageal, gastric and small bowel surgery	48.48 (10.81)	48.71 (10.83)	0.895
Head and neck thyroid parathyroid surgery	49.95 (28.01)	44.80 (27.69)	0.242
Hepatobiliary and Pancreatic surgery	64.89 (14.35)	57.99 (19.06)	0.009
Vascular artery and aorta, Vascular veins	59.98 (11.95)	51.02 (15.34)	< 0.001
Trauma of abdomen, chest, neuro, vascular	49.99 (14.28)	50.10 (13.44)	0.958
Plastic surgery and burn	61.39 (16.87)	57.87 (21.10)	0.237
Breast surgery Subspecialty Skin soft tissue surgery Colorectal and appendix surgery	57.56 (14.04) 51.66 (18.96) 61.08 (21.13) 49.62 (12.92)	53.19 (15.67) 52.88 (20.25) 59.72 (22.29) 47.85 (14.84)	0.062 0.692 0.691 0.416
**4** ^**th**^ **year residency**			
Esophageal, gastric and small bowel surgery	53.31 (12.68)	53.22 (11.77)	0.970
Head and neck thyroid parathyroid surgery	53.14 (24.26)	54.32 (29.19)	0.814
Hepatobiliary and Pancreatic surgery	71.62 (14.77)	69.53 (12.27)	0.433
Vascular artery and aorta, Vascular veins	60.09 (16.16)	59.59 (16.38)	0.872
Trauma of abdomen, chest, neuro, vascular	51.21 (13.02)	54.11 (12.11)	0.235
Plastic surgery and burn	64.02 (18.19)	64.88 (17.55)	0.804
Breast surgery Subspecialty Skin soft tissue surgery Colorectal and appendix surgery	63.30 (12.79) 54.66 (16.83) 59.13 (20.42) 55.68 (15.09)	59.30 (12.96) 49.54 (19.80) 67.56 (19.21) 54.68 (13.36)	0.107 0.142 0.030 0.719

When divided by the length of time that institutions have been accredited by the RCST (Table 3), there was no significant difference in test duration or overall scores between institutions accredited for more than seven years and those accredited for less than seven years. However, when analyzed by category, residents from institutions accredited for more than seven years tended to score higher in the HPB category. Looking at scores by residency year and category (Table 4), statistically significant differences were found only in the vascular category for first- and third-year residents and in the subspecialty category for third-year residents (*p* = 0.006, 0.018, and 0.025, respectively).

**Table 3:** Characteristic of training year

Parameters	Training year		*p*-value
	More than 7 years (*N* = 497)	Less than 7 years (*N* = 116)	
**Gender**,** n (%)**			0.664
Male	329 (66.20)	74 (63.79)	
Female	168 (33.80)	42 (36.21)	
**Resident year**,** n (%)**			0.429
1^st^	133 (26.76)	37 (31.90)	
2^nd^ 3^rd^ 4^th^	135 (27.16) 139 (27.97) 90 (18.11)	30 (25.86) 25 (21.55) 24 (20.69)	
**Average time to finished**			
Mean (SD)	169.76 (14.90)	170.49 (14.08)	0.629
**Examination (mins)**			
1^st^ year Residency 2^nd^ year Residency 3^rd^ year Residency 4^th^ year Residency	168.89 (15.19) 171.72 (12.32) 172.70 (11.35) 163.54 (20.16)	172.84 (9.60) 172.90 (8.74) 168.56 (18.66) 165.88 (18.56)	0.137 0.619 0.136 0.610
**Total score**,** mean (SD)**			
1^st^ year Residency 2^nd^ year Residency 3^rd^ year Residency 4^th^ year Residency	45.08 (7.12) 49.42 (8.30) 54.90 (7.64) 59.37 (6.85)	44.31 (6.09) 48.17 (11.07) 51.70 (8.28) 58.30 (6.83)	0.551 0.487 0.059 0.501
Scored by categories, mean (SD)			
Esophageal, gastric and small bowel surgery	47.11 (12.52)	47.37 (11.55)	0.837
Head and neck thyroid parathyroid surgery	45.75 (27.20)	41.11 (27.14)	0.098
Hepatobiliary and Pancreatic surgery	57.98 (18.30)	53.67 (19.25)	0.024
Vascular artery and aorta, Vascular veins	54.52 (15.07)	8.80 (15.90)	< 0.001
Trauma of abdomen, chest, neuro, vascular	48.34 (13.80)	49.05 (12.99)	0.618
Plastic surgery and burn	55.14 (19.96)	54.31 (20.66)	0.689
Breast surgery Subspecialty Skin soft tissue surgery Colorectal and appendix surgery	51.49 (16.60) 50.93 (18.69) 57.87 (22.56) 45.20 (15.30)	50.33 (17.82) 47.81 (18.76) 59.14 (22.29) 43.88 (14.70)	0.503 0.106 0.584 0.400

**Table 4:** Comparison scoring by training year year-by-year residency

Parameters	Training year		*p*-value
	More than 7 years (*N* = 497)	Less than 7 years (*N* = 116)	
	Mean (SD)	Mean (SD)	
**1** ^**st**^ **year residency**			
Esophageal, gastric and small bowel surgery	42.45(11.03)	43.88(12.81)	0.503
Head and neck thyroid parathyroid surgery Hepatobiliary and Pancreatic surgery Vascular artery and aorta, Vascular veins Trauma of abdomen, chest, neuro, vascular Plastic surgery and burn Breast surgery Subspecialty Skin soft tissue surgery Colorectal and appendix surgery	38.98 (26.56) 47.82(16.41) 50.03 (14.30) 44.63(13.98) 47.90(19.65) 43.27 (15.09) 48.88 (18.14) 51.43 (23.39) 37.54 (13.34)	32.46 (24.32) 43.58(16.79) 42.48 (15.28) 42.74 (10.53) 45.45 (17.76) 44.50 (19.35) 50.56 (18.09) 52.43 (23.74) 37.96 (11.40)	0.181 0.169 0.006 0.447 0.495 0.683 0.617 0.818 0.861
**2** ^**nd**^ **year residency**			
Esophageal, gastric and small bowel surgery Head and neck thyroid parathyroid surgery Hepatobiliary and Pancreatic surgery Vascular artery and aorta, Vascular veins Trauma of abdomen, chest, neuro, vascular Plastic surgery and burn Breast surgery Subspecialty Skin soft tissue surgery Colorectal and appendix surgery	45.64(13.09) 43.84(25.92) 54.03 (17.44) 52.50 (14.60) 48.20(13.47) 52.08(19.42) 48.20 (16.67) 48.46 (19.36) 58.52 (23.13) 41.09 (14.00)	47.92 (11.64) 42.17(28.84) 51.67 (19.13) 47.16 (13.45) 50.61(12.76) 48.82(21.83) 45.13 (18.02) 46.26 (16.28) 63.33 (20.40) 43.20 (15.13)	0.380 0.755 0.510 0.068 0.373 0.416 0.369 0.563 0.294 0.464
**3** ^**rd**^ **year residency**			
Esophageal, gastric and small bowel surgery	48.92 (10.95)	46.69 (9.78)	0.343
Head and neck thyroid parathyroid surgery Hepatobiliary and Pancreatic surgery Vascular artery and aorta, Vascular veins Trauma of abdomen, chest, neuro, vascular Plastic surgery and burn Breast surgery Subspecialty Skin soft tissue surgery Colorectal and appendix surgery	49.05 (28.18) 62.81 (16.31) 57.21 (13.96) 50.29 (14.22) 59.60 (18.90) 55.88 (15.21) 53.63 (19.01) 60.72 (21.59) 49.34 (13.56)	40.32 (25.58) 56.86 (19.17) 49.92 (14.13) 48.63 (12.00) 61.38 (18.80) 54.44 (13.10) 44.18 (20.44) 59.20 (21.97) 46.13 (14.83)	0.150 0.105 0.018 0.583 0.665 0.656 0.025 0.747 0.284
**4** ^**th**^ **year residency**			
Esophageal, gastric and small bowel surgery	53.41 (12.99)	52.78 (9.37)	0.825
Head and neck thyroid parathyroid surgery Hepatobiliary and Pancreatic surgery Vascular artery and aorta, Vascular veins Trauma of abdomen, chest, neuro, vascular Plastic surgery and burn Breast surgery Subspecialty Skin soft tissue surgery Colorectal and appendix surgery	53.52 (26.12) 71.43(14.11) 60.02 (16.15) 51.05 (12.28) 63.53 (18.22) 61.77 (12.95) 53.53 (17.30) 62.00 (20.12) 56.29 (14.03)	53.92 (27.05) 68.40 (12.67) 59.43 (16.60) 57.25 (13.29) 67.46 (16.47) 61.53 (13.20) 49.31 (21.10) 64.17 (21.25) 51.52 (15.37)	0.948 0.341 0.876 0.033 0.341 0.934 0.314 0.644 0.150

Finally, regional analysis showed no significant differences in overall performance between residents from different regions of Thailand, though some categories did show regional variations. For instance, third-year residents in the Northeast region scored significantly higher in the Vascular surgery category (66.00 ± 13.94) compared to their peers in other regions (*p* = 0.009) (Table 5).

**Table 5:** Comparison scoring by regional year-by-year residency

	Regional					*p*-value
	Central Mean (SD)	North Mean (SD)	East Mean (SD)	Northeast Mean (SD)	South Mean (SD)	
**1** ^**st**^ **year residency**						
Esophagus, small bowel, stomach	42.99(11.96)	42.67(11.54)	47.40(12.60)	41.52 (9.86)	42.05(11.59)	0.739^ANOVA^
Head and neck thyroid parathyroid	38.75(27.22)	29.98(21.76)	33.36(23.14)	36.98(26.53)	45.91(27.08)	0.478
Hepatobiliary and Pancreatic surgery	47.26(16.04)	46.59(16.29)	52.68(14.60)	47.54(17.72)	41.85(17.97)	0.467
Vascular artery and aorta, Vascular veins	50.40(13.72)	43.70(16.31)	47.89(13.53)	46.41(14.45)	50.15(17.74)	0.289 ^ANOVA^
Trauma of abdomen, chest, neuro, vascular	44.62(13.84)	42.93(13.63)	50.67(17.84)	42.41(10.71)	44.53(12.80)	0.809
Plastic, burn	47.11(20.91)	51.11(16.91)	39.14(20.62)	48.99(17.07)	44.25(17.98)	0.532
Breast	44.36(15.18)	39.66(16.22)	41.02(22.07)	43.97(17.04)	45.75(15.24)	0.625
Subspecialty	50.93(17.93)	49.52(17.73)	51.39(16.67)	47.74(19.76)	43.42(17.03)	0.554 ^ANOVA^
Skin soft tissue	55.06(24.19)	44.62(19.02)	55.56(19.44)	50.29(25.84)	47.37(21.30)	0.281 ^ANOVA^
Colo, appendix	37.30(13.16)	37.41(10.78)	33.51(13.13)	35.94(13.12)	44.46(12.96)	0.141 ^ANOVA^
**2** ^**nd**^ **year residency**						
Esophagus, small bowel, stomach	44.41(11.73)	46.56(17.33)	47.20(10.43)	50.13(13.49)	45.13(11.19)	0.282 ^ANOVA^
Head and neck thyroid parathyroid	43.37(26.23)	40.03(25.01)	45.28(30.31)	45.41(27.95)	44.00(26.22)	0.963
Hepatobiliary and Pancreatic surgery	51.37(16.85)	51.11(19.05)	49.79(14.08)	60.89(17.69)	56.33(20.64)	0.102
Vascular artery and aorta, Vascular veins Trauma of abdomen, chest, neuro, vascular	52.74(15.20) 47.54(13.25)	49.65(14.48) 52.10(12.33)	46.86 (7.81) 50.04(13.06)	50.03(15.85) 51.00(15.31)	53.30 (8.57) 43.67 (8.56)	0.658 ^ANOVA^ 0.278
Plastic, burn	50.10(19.02)	56.30(23.36)	65.49(19.63)	50.95(19.25)	45.35(18.70)	0.214
Breast	50.47(16.13)	49.61(12.06)	40.18(12.60)	41.18(21.32)	47.35(13.61)	0.052
Subspecialty	48.08(18.23)	45.84(20.06)	47.69(20.53)	45.11(18.25)	59.30(19.86)	0.218 ^ANOVA^
Skin soft tissue	58.65(23.32)	61.00(17.74)	62.22(30.73)	57.06(22.09)	66.15(22.19)	0.767 ^ANOVA^
Colo, appendix	39.79(13.89)	40.86(12.23)	34.12(10.66)	46.13(13.87)	46.92(18.39)	0.052 ^ANOVA^
**3** ^**rd**^ **year residency**						
Esophagus, small bowel, stomach	49.49(10.98)	48.55 (9.53)	52.37(12.34)	46.59 (9.25)	46.39(12.54)	0.502 ^ANOVA^
Head and neck thyroid parathyroid	50.19(28.85)	43.68 (25.21)	35.73(20.62)	46.94(30.68)	45.96(24.39)	0.863
Hepatobiliary and Pancreatic surgery	61.97(16.41)	66.10 (16.00)	66.36(12.36)	55.47(17.47)	65.71(18.38)	0.126
Vascular artery and aorta, Vascular veins	58.03(14.22)	57.30 (12.52)	66.00(13.94)	48.99(14.47)	53.86(11.56)	0.009 ^ANOVA^
Trauma of abdomen, chest, neuro, vascular	48.84(13.59)	54.68 (10.40)	66.84(11.29)	49.02(14.24)	46.91(14.38)	0.009
Plastic, burn	57.97(19.33)	62.77 (19.33)	79.40(12.72)	58.36(21.62)	60.97(13.60)	0.071
Breast	56.72(15.73)	55.97 (12.85)	56.01 (9.62)	53.07(14.02)	54.57(16.09)	0.815
Subspecialty	49.63(17.19)	48.86 (19.95)	76.20 (8.90)	52.78(21.71)	56.94(22.25)	0.006 ^ANOVA^
Skin soft tissue	59.55(22.69)	61.11 (16.05)	74.29(19.02)	58.67(20.97)	61.90(22.72)	0.506 ^ANOVA^
Colo, appendix	49.12(13.33)	49.63 (12.19)	60.53(12.96)	43.36(15.03)	51.02(12.96)	0.032 ^ANOVA^
**4** ^**th**^ **year residency**						
Esophagus, small bowel, stomach	53.49(12.66)	52.15 (11.99)	40.95(12.09)	53.58(11.16)	54.88(13.84)	0.679 ^ANOVA^
Head and neck thyroid parathyroid	55.79(27.85)	42.89 (18.90)	39.30(35.36)	55.53(25.58)	51.99(24.55)	0.406
Hepatobiliary and Pancreatic surgery	71.93(14.00)	68.64 (9.68)	44.25(18.17)	70.89(14.23)	71.55(12.43)	0.259
Vascular artery and aorta, Vascular veins Trauma of abdomen, chest, neuro, vascular	63.00(16.32) 50.87(12.02)	52.75 (14.51) 56.20 (13.33)	53.60(21.92) 60.50(13.15)	59.53(15.21) 56.13(13.15)	52.83(16.21) 45.77(12.07)	0.122 ^ANOVA^ 0.274
Plastic, burn	66.94(16.95)	60.95 (21.24)	67.37 (0)	62.26(18.95)	58.27(17.48)	0.539
Breast	62.63(12.89)	59.16 (12.29)	46.00 (0)	62.40(13.63)	60.83(13.06)	0.352
Subspecialty	53.23(18.70)	56.74 (19.30)	37.50 (5.94)	52.41(15.06)	47.75(21.75)	0.577 ^ANOVA^
Skin soft tissue	64.19(19.55)	55.38 (24.70)	50.00(42.42)	60.77(19.98)	67.27(16.18)	0.471 ^ANOVA^
Colo, appendix	56.90(14.21)	49.49 (15.88)	45.15 (6.86)	55.03(13.94)	55.45(15.21)	0.425 ^ANOVA^

## Discussion

Among the residents from the two types of institutions compared, only 1st-year residents showed a statistically significant difference in the time taken to complete the examination, with those in public health training institutions taking more time than those in medical training institutions. This difference may be attributed to varying approaches to examination preparation. When analyzing overall scores by category, differences were noted in the HPB (Hepatobiliary and Pancreatic) and vascular system sections between the institutions. These differences might be due to residents in medical schools encountering more complex cases and rare diseases compared to those in public health institutions. Furthermore, some public health institutions may lack access to, or have limited availability of, vascular and HPB surgeons, which restricts them to basic surgical procedures and interventions due to limited resources. Consequently, complex major operations and advanced interventions in HPB and vascular surgery may not be performed in these public health institutions.

These findings can also be explained by the fact that most medical schools are organized into specialist divisions that are organ-oriented. As residents rotate through each division, they gain experience by encountering a broader range of cases. In contrast, examinees from public health training institutions scored higher in the esophagus, small bowel, stomach, and trauma sections, as the majority of patients in these institutions fall into these categories. Additionally, medical training institutions in Thailand primarily serve as tertiary care centers, leading to a lower number of in-hospital patients admitted for common surgical conditions such as peptic ulcer perforation, small bowel obstruction, and trauma. The cutoff point used in this study to distinguish between different levels of surgical residency training experience was 7 years, as approved by the RCST. This cutoff was chosen because the MCQ examination period includes several institutions that have been approved for training, with each institution undergoing evaluation every four years.

In this study, there are no specific criteria for passing each residency year. However, scores and average scores are collected and used in conjunction with the standard deviations at the national and institutional levels as a reference for self-evaluation. In-training evaluations during training sessions have been found to correspond with the scores obtained in actual board exams in the future [13–16]. Therefore, if an examinee does not score well in a particular area, they can focus their development on specific categories to improve themselves.

The study also examined the impact of the length of time an institution has been accredited as a training center. It was observed that institutions with more than seven years of experience tended to score higher in specific categories like HPB surgery, suggesting that the duration and stability of a training program may contribute to better outcomes in certain specialized areas. However, this difference was not universally significant across all categories, indicating that the length of accreditation may not have a substantial impact on overall medical knowledge.

Despite the differences in training environments, the overall conclusion of the study was that the medical knowledge as assessed by the MCQs did not significantly differ between residents trained in medical school-based and public health-based institutions. This finding highlights the effectiveness of the standardized training and evaluation system accredited by the Royal College of Surgeons of Thailand (RCST), which ensures that all institutions, regardless of their focus or resources, are capable of providing equivalent levels of training and education to their surgical residents. The accreditation system by a central organization that is not involved with the institution is very important and key to success in controlling the quality of the training system under different environments.

One potential next step for this study could be to compare the scores of each individual test-taker across different categories to see if there has been any significant improvement or decline in certain areas. This could include analyzing overall scores as well as scores from specific sub-categories or sections of the exam.

The primary strength of this study is that it represents the first to examine and report on the relationship between institution type and MCQ scores—categorized as either medical school-based or public health hospital-based training institutions—while providing a detailed analysis of the differences between these categories.

## Conclusion

the study shows that there is no significant difference in the MCQs exam results between residents trained at medical school-based institutions and those at public health-based institutions. Additionally, the location of the training center, whether in the Central, Northern, Eastern, Northeastern, or Southern regions of Thailand, does not affect the overall mean scores, which reflect the medical knowledge of surgical residency. This finding highlights the effectiveness of the standardized training and evaluation system accredited by the Royal College of Surgeons of Thailand (RCST), ensuring that high-quality surgical education is delivered uniformly across diverse settings, regardless of resource levels.

## Data Availability

No datasets were generated or analysed during the current study.
